# A kind of integrated serial algorithms for noise reduction and characteristics expanding in respiratory sound

**DOI:** 10.7150/ijbs.33274

**Published:** 2019-07-21

**Authors:** Fei Meng, Yixuan Wang, Yan Shi, Hongmei Zhao

**Affiliations:** 1 School of Automation Science and Electrical Engineering, Beihang University, Beijing 100191, China; 2 The State Key Laboratory of Fluid Power Transmission and Control, Zhejiang University, Hangzhou 310058, China; 3 Department of Pulmonary and Critical Care Medicine, China-Japan Friendship Hospital

**Keywords:** respiratory sounds, de-noising algorithm, band-pass filter, wavelet, adaptive filter

## Abstract

The computer‑based lung respiratory sound analysis, which can provide more information about the condition of lung station, has achieved a great development in recent years. However, the external noise in respiratory sound signal is a large restriction to the further promotion of this technique. In this paper, a kind of serial integrated de-noising algorithms which consist of a FIR band-pass filter and a modified wavelet filter and an adaptive filter, is proposed to suppress the noise in respiratory sound signals. The design of this kind of filter and its practical application are studied. The practical application in de-noise of the lung sound shows that this filter has a good de-noising effect and a good performance in outstanding the acoustic characteristics.

## Introduction

Respiratory sounds can provide much information about the condition of lung station, and they are a kind of nonlinear and non‑stationary signals. The auscultation of lung sounds provides a point of reference for the diagnose of respiratory diseases, like pneumonia, bronchitis and sleep apnea 1. Most of the auscultation of lung sounds and diagnose of respiratory disorders, traditionally, depend on the medical skills and diagnostic experience of physicians. In the last decades of years, the computer‑based respiratory sound technique has achieved a great development to serve as a more effective and general tool in the diagnose of lung abnormalities 2.

In recent years, the electronic stethoscope has been widely used in the field of lung sound analysis, with its unique feature of digitalization and high sensitivity similar to the traditional stethoscope. Some mature products like, 3M Littmann, have emerged on the market. However, the external interference which is produced by high-frequency and low-frequency environmental noises, human voices and heart sounds, is a large restriction to the lung sound collected by electronic stethoscopes in the field of auscultation and further research. Hence, the noise reduction becomes a crucial step of lung-sound signals processing.

The traditional approach to the problem is to use a linear high-pass filter with a cutoff frequency from 50Hz to 150Hz. However, because the frequency of lung sounds is in the range of 20Hz to 1600Hz 3, overlapping the frequency of heart sounds varying from 20Hz to 150Hz 4,5. Therefore, a single high-pass linear filter is bound to cause the distortion of the respiratory sounds, which is not effective in the problem. The 3M Littmann electronic stethoscope shows good de-noising effect in mid-frequency and high-frequency bands. However, some components in overlapping frequency region in low frequency domain are degraded in the signals measured by the products, which produces a certain degree of distortion of lung sounds.

To achieve better performance on de-noising, researchers have developed two basic approaches for noise reduction: the wavelet shrinkage de-noising and the adaptive filtering algorithm.

Thanks to the mature technology and low-complexity, wavelet transform is widely used in the medical-signal process, for example 6,7,8,9. In the field of respiratory sound de-noising, the wavelet shrinkage de-noising also plays a significant role. M Bahoura et al 10 proposed a new technique for de-noising lung sounds based on the wavelet packet, which can reduce the noise without distortion of the original sound. Hossain I et al 11 investigated the spectral characteristics of the lung sound signals through wavelet transform filters and found that the filters reduce the lung sound average power greatly over the whole frequency range.

The adaptive filter, which is used by many researchers, is another de-noising algorithm in respiratory sound. Sankar et al 12 compared the de-noising effect of several different types of adaptive filters applied on the model respiratory sound with second order Auto Regressive process. However, the adaptive filter is usually utilized to reduce heart sounds, for the good de-noising effect, without significantly affecting the components of lung sounds in low-frequency region. This method applied in heart sound reduction is generally considered to be proposed by Iyer et al 4 to use an adaptive filter with an 'augmented electrocardiogram (ECG)' signal as reference signal to reduce heart sounds by 50-80 percent. But the import of external ECG signal makes the filter inconvenience to use. Hence, Kompis et al 13 modified the algorithm model to get an adaptive filter which needs only a single microphone as input. Heart noises (HN) can be reduced moderately by 24% to 49% using this method. To improve the HN reduction, an adaptive filter combined with the fourth-order statistics (FOS) was proposed by Hadjileontiadis and Panas 14. The HN reduction of this algorithm is more than 90%, at the expense of algorithm complexity. In addition, Hadjileontiadis and Panas^15^ proposed a new type of adaptive filter based on wavelet transform (WT), combining the method of an adaptive filter and wavelet composition, which has an efficient reduction of heart noises.

In this paper, a Type I Chebyshev band-pass filter with a pass-band from 20Hz to 1600Hz is firstly proposed to reduce the noise of the lung respiratory sounds under the low frequency and high frequency environment. Next, we put forward a unification wavelet threshold de-noising method to reduce the environmental noises further and to mitigate the second heart sound and human voice. Last, an adaptive noise cancellation filter is designed to reduce the heart sounds. The following sections will discuss the configuration and the practical experiment practical in the experiment signals collected from eleven patients in the hospital.

### Lung sound signal acquisition

**Figure 1:**
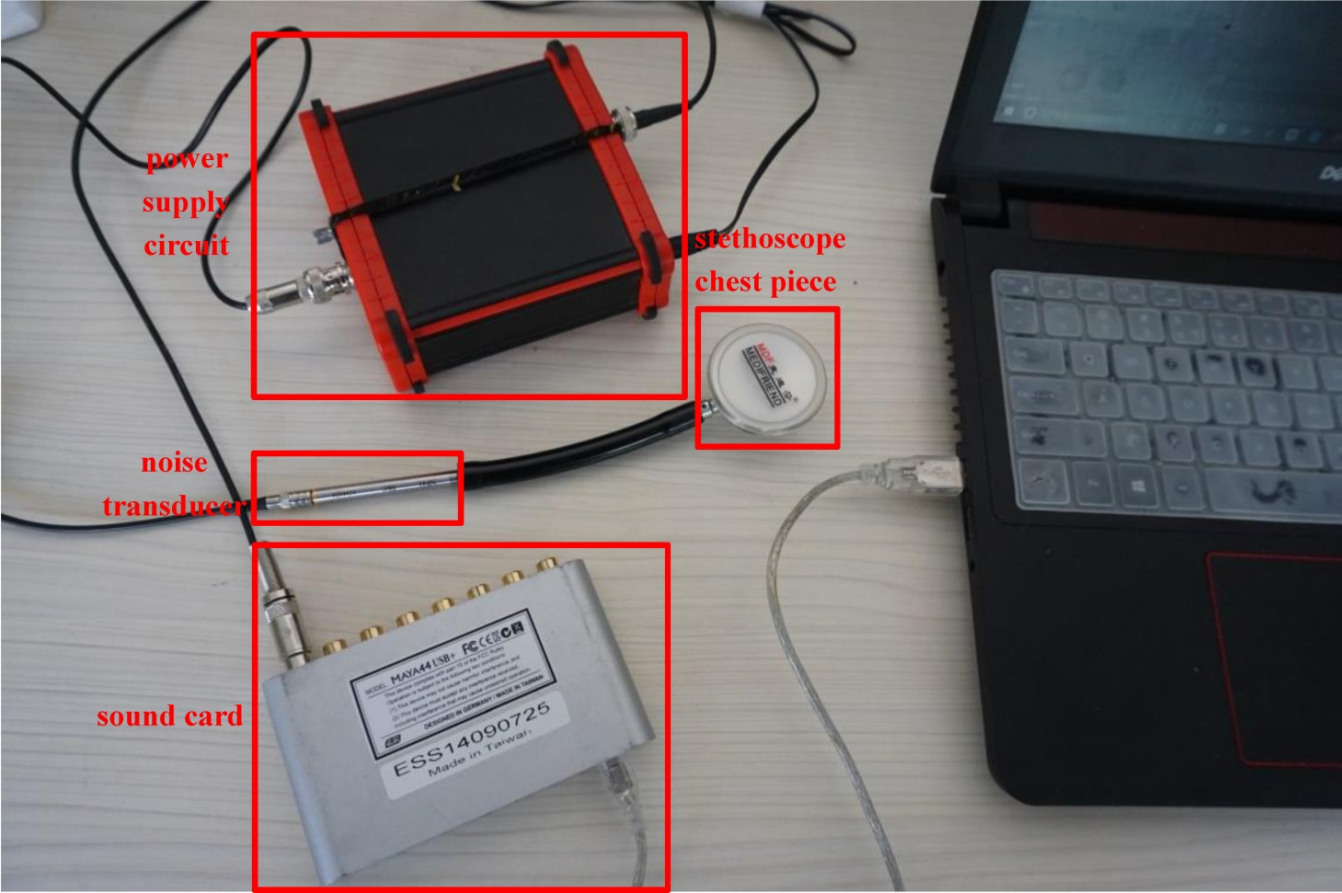
respiratory sound recorded equipment

**Figure 2:**
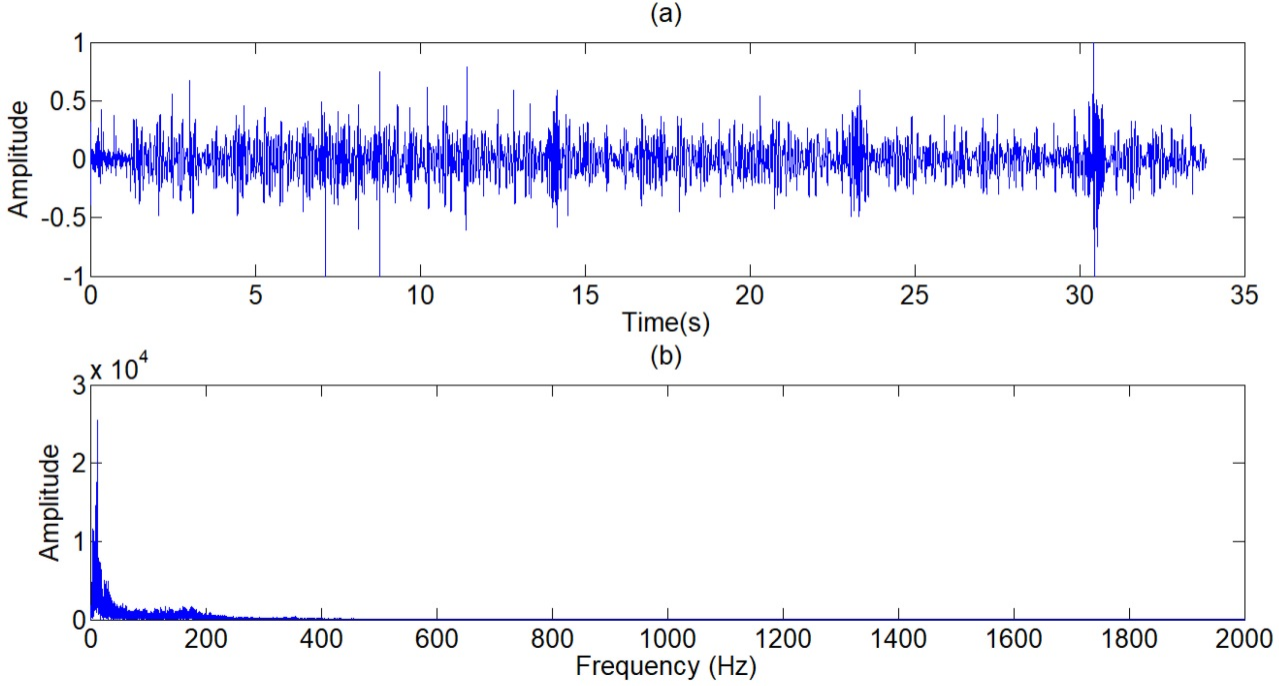
(a) Standard signal, (b) Frequency spectrum of standard signal.

The lung sound signals are recorded by the equipment designed and assembled by the research team, as shown in Fig 1. The main component of the set of sound measurement equipment is a highly sensitive noise transducer fixed in a short piece of plastic tubing connected to a stethoscope chest piece. The sensitivity of transducer is -36dB and the frequency response is 5Hz to 100kHz. The input impedance is more than 5G ohms and the output impedance is less than 110 ohms. The other part of the system is consisted of a power supply circuit of the transducer, a 32-bit sound card used to collect the digital signal and a computer for data storage. The sampling frequency of the sound card is 48000Hz. The study was conducted in the hospital on eleven patients with moist rales, dry rales and normal lung sounds. An example of lung sounds is used to analyze the de-noising algorithm as shown in Fig 2(a), which is defined as the standard signal in this paper, while the other samples are used to verify the effect of the algorithm and adjust algorithm parameters. The main parameters of the signal device are shown as follows.

### De-noising Algorithm

**Figure 3:**
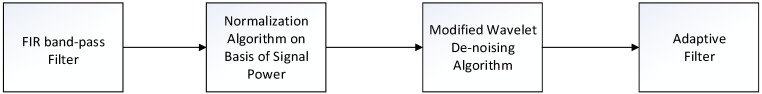
Configuration of the proposed serial integrated algorithm

The structure of the overall serial integrated algorithms is shown in Fig 3, combined with a FIR band-pass filter, a modified wavelet filter and an adaptive filter. The type I Chebyshev band-pass filter is designed to decrease the high frequency and low frequency environmental noises. The modified wavelet filter can reduce the environmental noises further and mitigate the second heart sounds and human voices. The least-mean-square (LMS) adaptive filter with the modified reference signal is used to decrease the influence of the heart sounds. The principle of the serial integrated filter is introduced in this chapter.

### Type I Chebyshev band-pass filter

**Figure 4:**
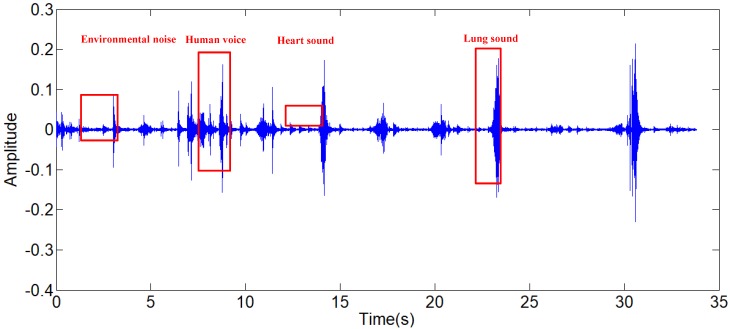
Output signal of FIR filter

Influenced by the environment factors, device factors and physical factors, the noise of lung sounds collected is concentrated in the low-frequency and high frequency. To eliminate the main part of low-frequency and high-frequency noises, we choose a type I Chebyshev band-pass filter for ensuring a steep roll-off frequency. The frequency spectrum (see Fig 3.b) shows that the frequency of original signals varies from 0Hz to 500Hz. For retaining the whole frequency domain of the lung sounds, the pass-band of FIR filter is set from 15Hz to 1700Hz. The output of the filter is shown in Fig 4.

### Modified wavelet threshold de-noising method

#### Wavelet decomposition

The wavelet transform is defined as:


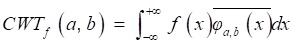
(1)

where *a* is the dyadic dilation, *b* is the dyadic position, and the wavelet mother function*φ*(*x*) is defined as:


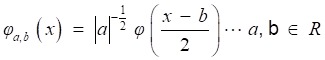
(2)

The discrete wavelet transform (DWT) is


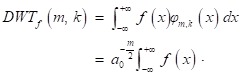



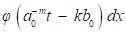
(3)

if the dyadic dilation *a*_0_=2 and the dyadic position *b*_0_=1, the DWT is called 2-band wavelet transform.

**Figure 5:**
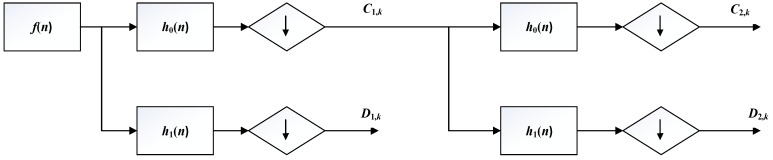
Structure of MALLAT algorithm

But the DWT still has high calculation complexity. Figure 5 shows the process of MALLAT algorithm which is a simple algorithm of wavelet decomposition. The signal *f*(*n*) to be decomposed is filtered by high-frequency filter *h_0_*(*n*) and low-frequency filter *h_1_*(*n*)*.* Then the filtered signals are down-sampled to get the wavelet coefficients 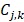 and approximation coefficients 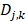 where *j* and *k* are respectively the dyadic dilation and dyadic position. The step of ↓ means the process of down-sampling. In the next layer of wavelet decomposition, the approximation coefficients of former layer are transformed as the signal to be decomposed and processed in the same way. The relationship of the two filters is


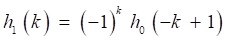
(4)

In the MALLAT algorithm, the signal is firstly through periodic extension, to eliminate the boundary effect, as


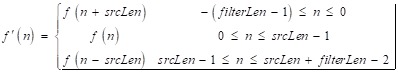
(5)

where *srcLen* is the length of the signal, *filterLent* is the length of the filter *h*_o_ and *h*_1_.

Then the signal is filtered by the high frequency filter and low frequency filter to get the wavelet coefficients and approximation coefficients. After the filters, the signal is down-sampled and a signal with half of the length is gotten.


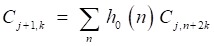
(6)


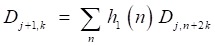
(7)

After one step of decomposition, the approximation coefficients are processed in the same way to get wavelet coefficients and approximation coefficients in the next layer. After *n* steps of iteration, the signal is decomposed into *n* groups of wavelet coefficients and one group of approximation coefficients. The wavelet reconstruction is the inverse operation of wavelet decomposition.

### Wavelet threshold de-noising algorithm

The concept of the wavelet transform de-noising was firstly proposed by Hadjileontiadis 14,15 and Panas 16, assuming that the wavelet coefficients of the “nonstationary” part is much lower than that of “stationary” part. Among the wavelet transform de-noising algorithms, the most widely used one is the wavelet threshold de-noising algorithm, which was firstly proposed by Donoho^17^, ^18^. A contaminated signal with finite length is assumed to be expressed as:


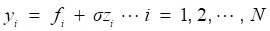
(8)

where *f_i_* is the desired signal, *z_i_* is the Gaussian noise with zero mean and variance *σ^2^,* which is independent from the desired signal *f_i_*.

Transform the noisy signal *y_i_* by discrete wavelet transform (DWT). Let *W* be a left invertible wavelet transformation matrix of the DWT 19. Then the Eq can be written as


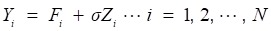
(9)

where *Y=Wy* is the wavelet coefficients of noisy signal *y_i_*, *F=Wf* is the wavelet coefficients of the uncontaminated signal *f_i_*, *Z=Wz* is the wavelet coefficients of noise signal *z_i_*. The basic idea of wavelet threshold de-noising is to zero the wavelet coefficients belonging to the noise *Z*, while keep the wavelet coefficients belonging to the useful signal *F*, and the filtered signal approximates the desired signal *f_i_*.

The wavelet thresholding consists of two methods: hard thresholding


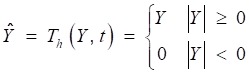
(10)

and soft thresholding.


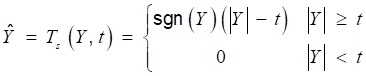
(11)

where *Ŷ* is the estimation of wavelet coefficients *Y*. The filtered signal *x* can be obtained by inverse DWT:


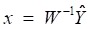
(12)

Several selection methods of threshold value play an important role in wavelet de-noising. Donoho proposed the thresholding rule 17:


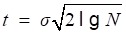
(13)

where *N* is the number of sampled points. While Guoxiang^18^ et al proposed the thresholding rule


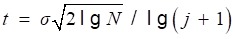
(14)

where *j* is the DWT scale. However, sound signals are highly sensitive to the thresholding value selection. An excessive thresholding value can lead to the distortion of sound signals, while a small thresholding value may not achieve a good filtering result. Therefore, the selection of thresholding values in this research cannot depend on a single fixed formula.

**Figure 6:**
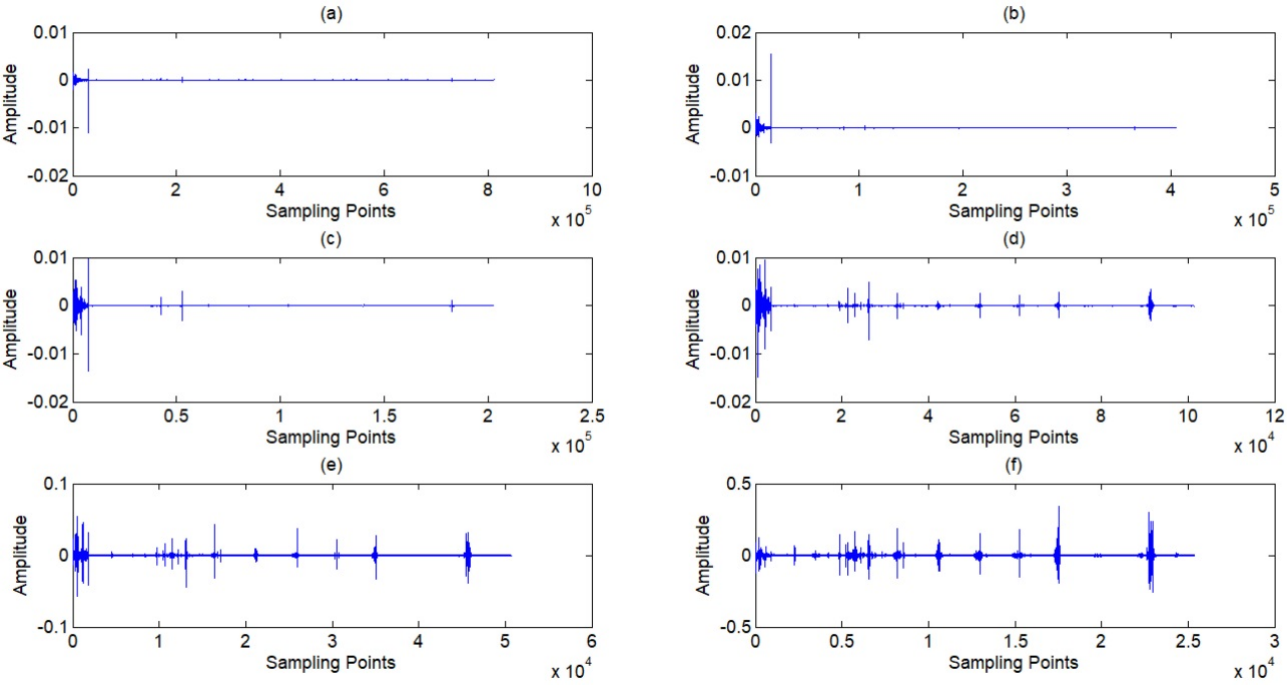
** (a) ~(f)** Wavelet coefficients of standard signal CD1~CD6, **(j)** Approximation coefficients of standard signal CA9.

In this research, a *coif2* wavelet basis is chosen. The signal *s* is decomposed to 9 layers, as shown in Fig 6, and nine sub-signals *s_i_* is reconstructed by each layer of wavelet coefficients. The relationship of the original signal *s* and sub-signals *s_i_* is given by:


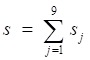
(15)

**Figure 8:**
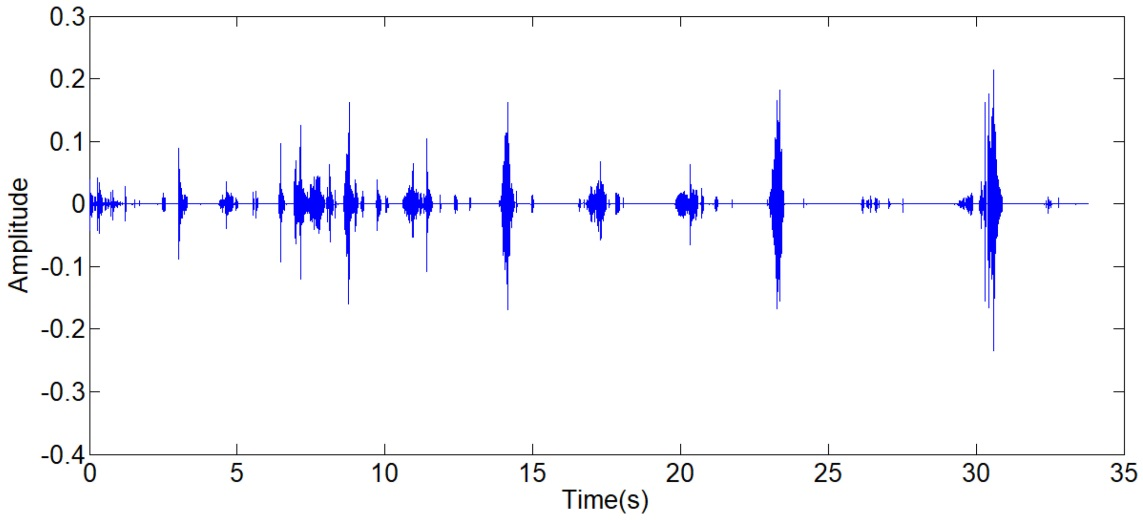
Reconstructed signal of wavelet filter

At the low resolutions (layer 1 and 2), there are only coefficients belonging to the noise at the high frequency domain. Therefore, the hard threshold method can be used to eliminate the high frequency noises directly. At most resolutions (layer 3,4,5,6,9), the proportion of useful signal components is higher, so the soft threshold method is chosen to avoid pseudo Gibbs phenomena. The selection of threshold value, as shown in Table 1, depends on the repeated adjustment based on the low distortion and high de-noising effect rather than any fixed formula. The majority components of the lung sound signal concentrate in the layer 7 and 8. A small deviation between the threshold value and ideal value may lead to a great distortion. Therefore, to ensure the low distortion of lung sound, the sub-signal is not processed by threshold de-noising filtered but the type I Chebyshev band-pass filter with the band-pass from 20Hz to 1600Hz to eliminate the noises beyond the frequency band. The reconstructed signal is shown in Fig 8, in which, the high-frequency and low-frequency noises, human voices and second heart sounds are largely reduced.

**Figure 9:**
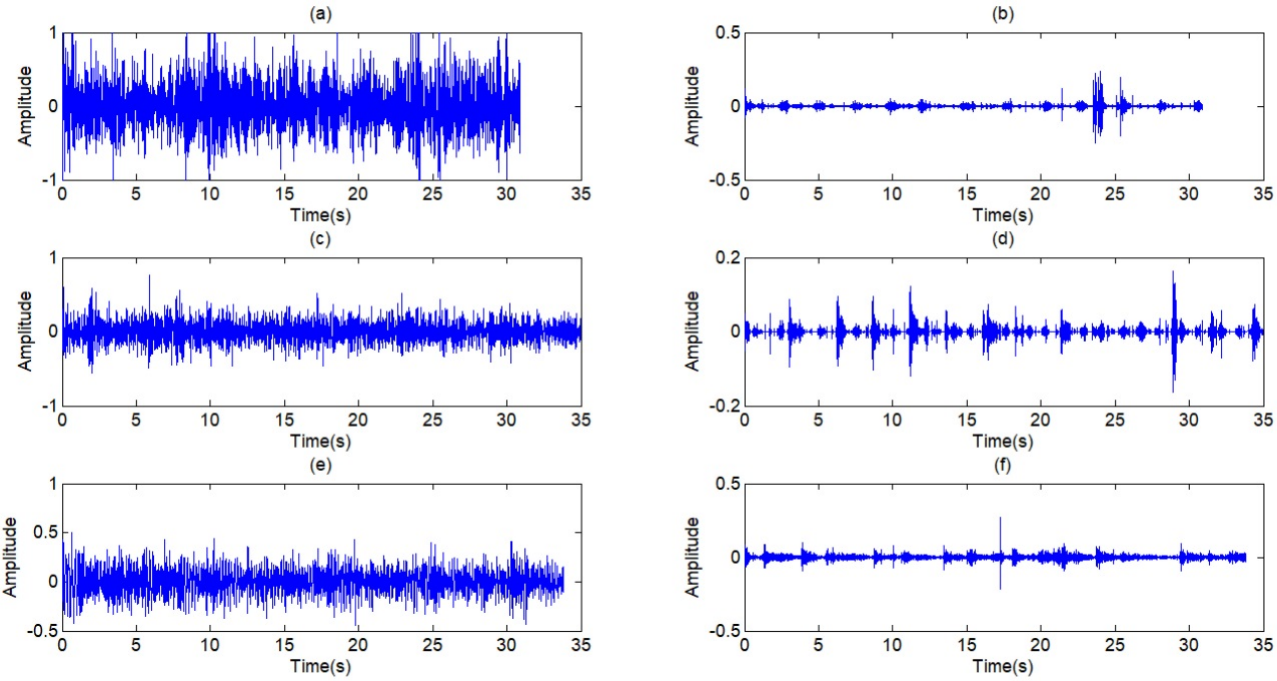
(a),(c),(e) The other three typical respiratory sounds, (b),(d),(f) The filtered signals of the three signals

The threshold value is determined on the basis of the standard signal in this paper. However, the signal strength has a considerable difference with the standard signal for the different patient situations and measurement environments, which leads to the different selection of threshold value. The phenomenon limits the generalization of the algorithm among different signals collected. To solve the problem, a normalization method based on the power of signals is proposed. The power of a discrete signal is given by


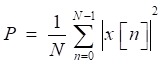
(16)

where, *x*[*n*] is the sampling points of the signal, *N* is the length of signal acquired. The original signal is normalized by


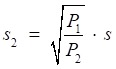
(17)

where, *P*_1_ is the power of standard signal. s is the signal to be processed. *P*_2_ is the power of the signal to be processed. Because the arrangement can reduce the effect of environmental noises on the power of signal, the step of normalization is arranged after the step of Chebyshev band-pass filter, as shown in Fig 3. Through this method, the strength of original signals is set to the similar magnitude of the standard signal, and the threshold value can be generalized on the signals with different signal strengths. There are three other signals acquired shown in Fig 9, which have similarly favorable de-noising effect. The first signal and the third signal belong to dry rales. The second signal belongs to the normal lung sound.

### Adaptive filter

**Figure 10:**
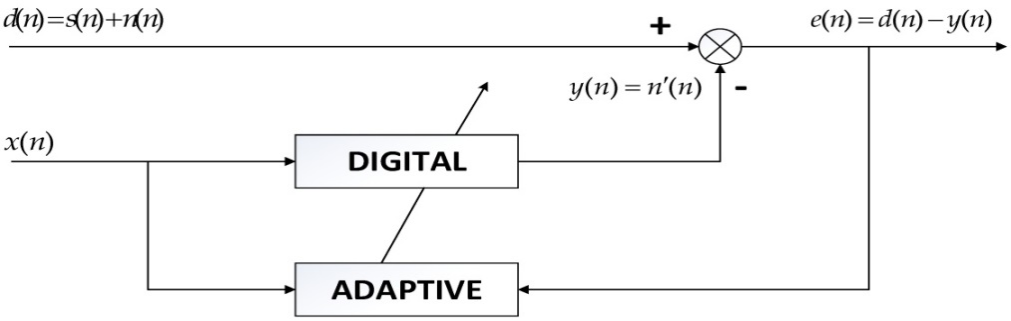
Principle of adaptive filter

Because the frequency of heart sounds overlaps with the low-frequency component of lung sounds, the filtering algorithms in frequency domain are not effective in this situation. The main approach in recent years to the problem is the adaptive filter. In this paper, a least-mean-square (LMS) adaption algorithm referring to 7,16,21 is modified to reduce the first heart sound. The block diagram of the algorithm is shown in Fig 10.

The obtained signal *d*(*n*) is the output signal of the wavelet threshold filter containing the desired the pure lung sound signal *s*(*n*) and the first heart sound *n*(*n*) as inference. The reference signal *x*(*n*), which is linearly related with the inference signal *n*(*n*), is obtained from the signal *d*(*n*). The estimated signal *n'*(*n*) is calculated through the Finite Impulse Response (FIR) filter by the reference signal *x*(*n*). The bias signal *e*(*n*) is obtained by the estimated signal *n'*(*n*) from the corrupted signal *d*(*n*). The coefficients of FIR filter are adjusted by the bias signal on cost function *ξ*(*n*) to make the estimated signal *n'*(*n*) more close to the inference *n*(*n*). In this way, the bias signal *e*(*n*) as the output of the filter can be more closed to the desired signal *d*(*n*).

The cost function of LMS adaptive filter to get a desired estimation of the inference *n(n)* is the minimization of mean-square error 16, which is defined as:


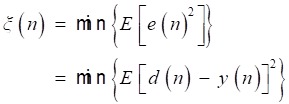
(18)

And the algorithm adjusts the taps of FIR filter by Eq (19).


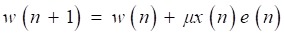
(19)

where *w*(n) is the tap of FIR filter, μ is step size which is chosen as 0.01. Referring to 4, from Fig 10, we can write


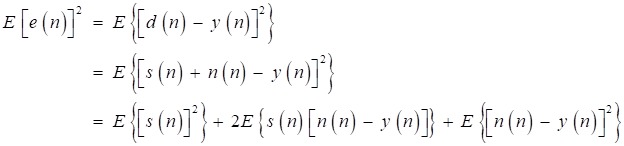
(20)

The first term *E*{[*s*(*n*)]^2^} is determined when the desired signal *s*(*n*) is fixed. Assuming that the inference signal and the desired signal are independent, and the second term *2E*{s(*n*)[*n*(*n*)-*y*(*n*)]} can be expressed as:


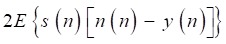

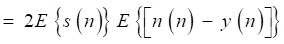
(21)

where *E*{[*n*(*n*)-*y*(*n*)]} is reduced to zero with iterations to run, and the second term is zero. Hence, the minimization of the mean-square error *E*{[*s*(*n*)]^2^} is equivalent to the minimization of the third term *E*{[*n*(*n*) -*y*(*n*)]^2^}*.* Therefore*,* the inference *n*(*n*) approximates the estimated signal *n'*(*n*), and the basis signal *e*(*n*) approximates the desired signal *s*(*n*). That is the basic mechanism of the LMS adaptive filter for heart-noise reduction.

The obtainment of reference signal *x*(*n*) is summarized as follows:

- On the basis of the estimation of the normal breath period, the signal is intercepted by the epoch window with the duration *N_e_*. The elements of the first column of two-dimensional vectors *MAX*{*maxv_1_*, *maxt_1_*; *maxv_2_*, *maxt_2_*;*…*; *maxv_m_*, *maxt_m_* } and *MIN* {*minv_1_*, *mint_1_*; *minv_2_*, *mint_2_*; ...; *minv_m_, mint_m_* } are the positive peak values and negative peak values of each window, while the elements of the second column are the corresponding time, and *m* is the number of windows.
- This step is to confirm whether the peaks are caused by heart beats or breath. Firstly, sort the positive peak values from large to small to get an ordered vector *OMAX* {*omax_1_*,*omax_2_*,*…*,*omax_m_*}, whose mean value is *MAX_ave_*. Secondly, find the element *omax_i-1_*, which is greater than 1.5∙*MAX_ave_*, while the following element, *omax_i_*, is less than 1.5∙*MAX_ave_*. The elements *omax_i+1_*, *omax_i+2_*,*…*, *omax_m_*, which are less than *omax_i_*, can be regarded as the peaks caused by breath or caused by the first heart sounds which are too small to be ignored. To avoid the phenomenon that the positive peak and the negative peak in the same window are caused by two different heart sounds on the edge of the window, the interval of the positive peak and negative peak in one window need to be checked. If the interval |*maxt_j_-mint_j_*| is less than a fixed value *N_t_*, the positive peak and negative peak can be supposed to be from the same heat beat, and the time of positive peak *maxt_j_* can be regarded as the time of heart beats.
- After confirming the time of heart beats, copy the original signal in the interval of [*maxt_j_*-*time_heart_*, *maxt_j_*+*time_heart_*], where *time_heart_* is half of the estimated heart beat times, while other regions of the signal are set to zero, and the signal obtained can be considered as the reference signal.

**Figure 11:**
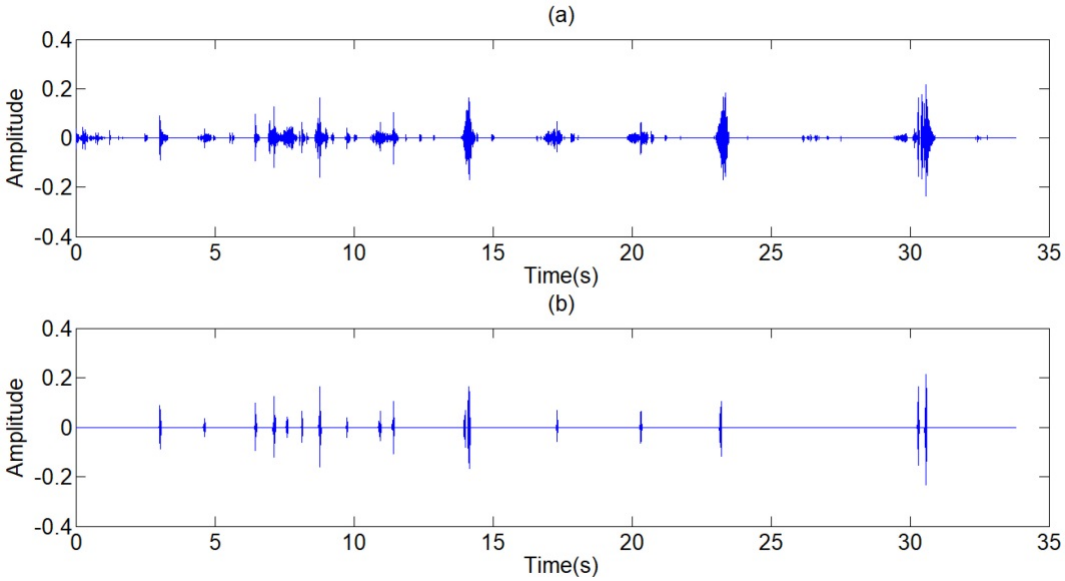
(a),(c),(e) The other three respiratory sounds, (b),(d),(f) The filtered signals of the three signals

The reference signal of the standard signal is shown in Fig 11.

**Figure 12:**
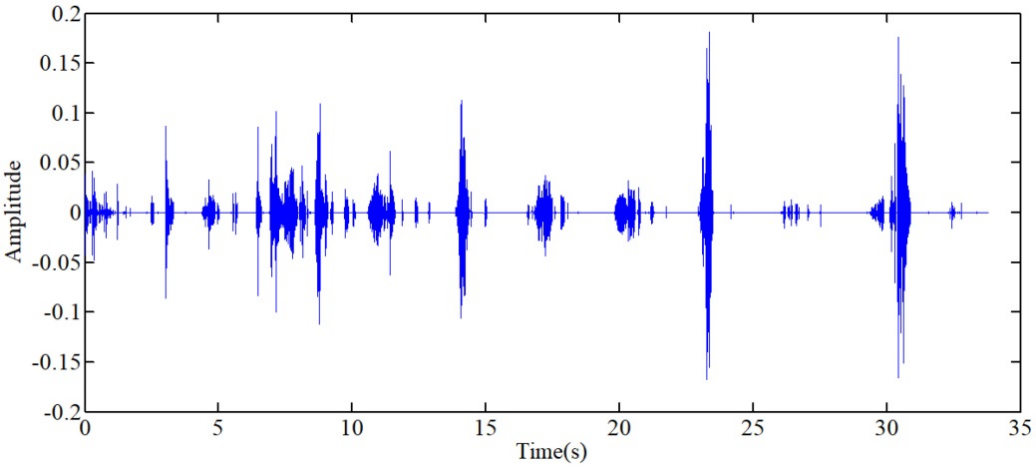
Filtered result of standard signal

**Figure 13:**
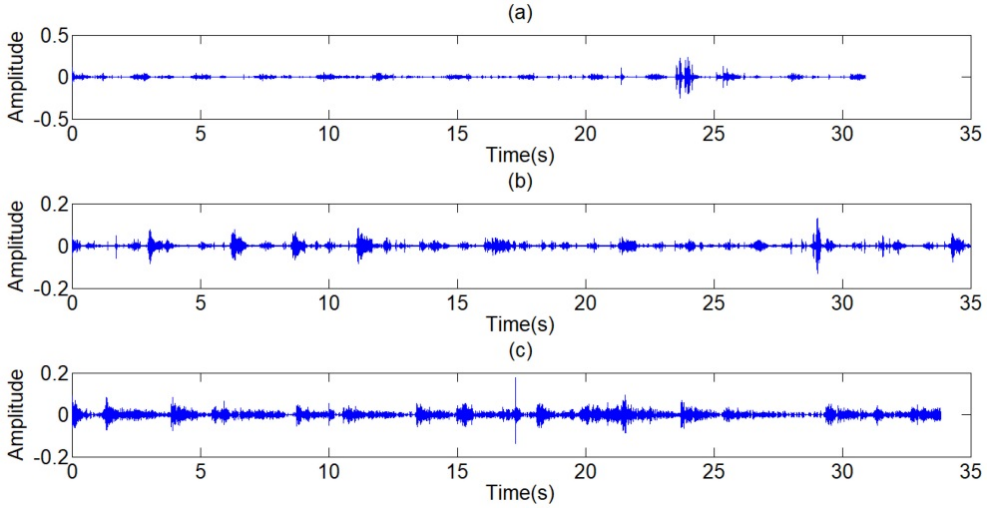
Filtered result of other typical signals

**Figure 14:**
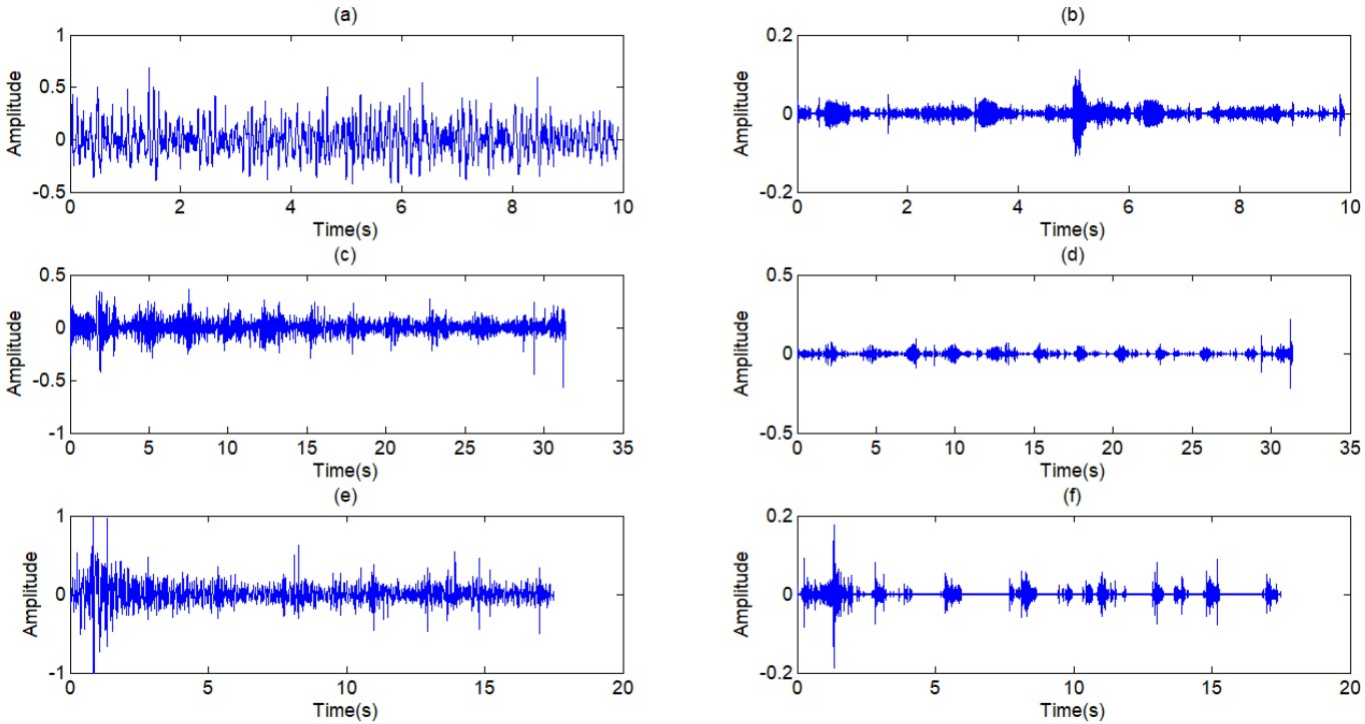
(a),(c),(e) Other three respiratory sounds, (b),(d),(f) The filtered signals of the three signals

**Figure 15:**
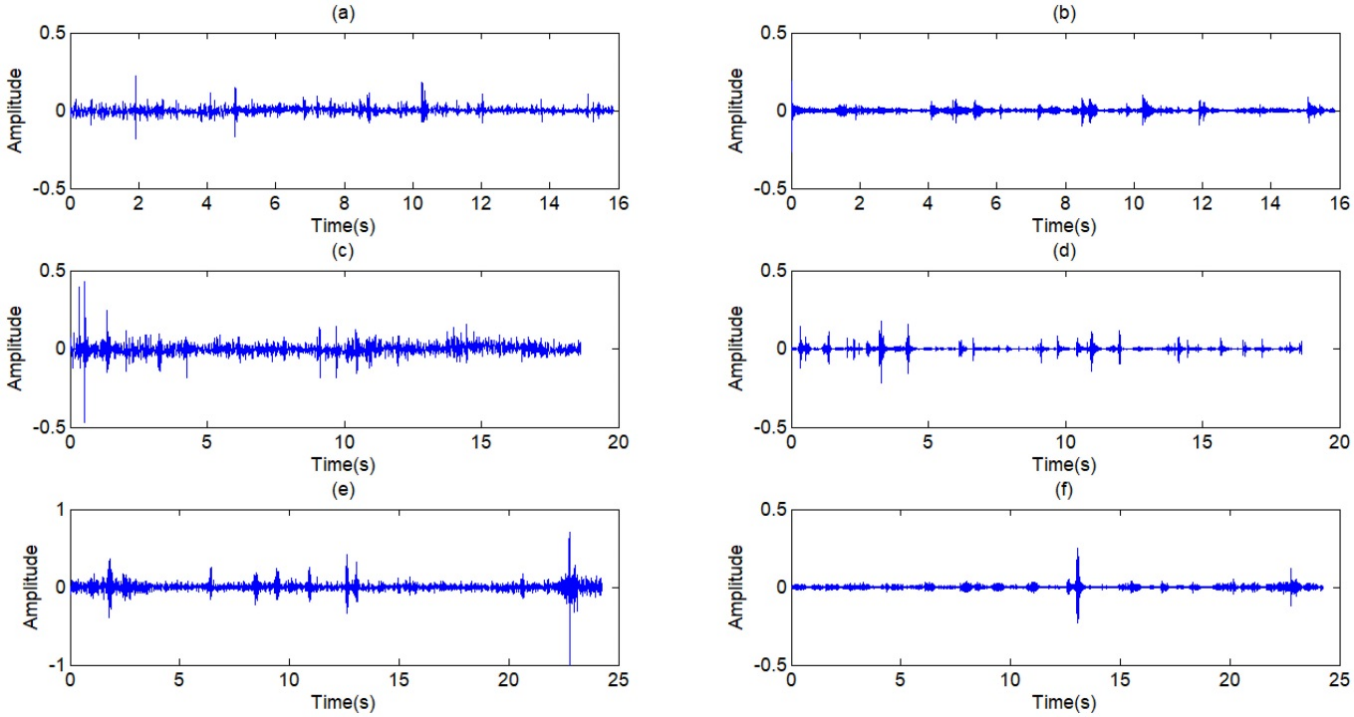
(a),(c),(e) Another three typical respiratory sounds, (b),(d),(f) The filtered signals of the three signals

The filtered results of the standard signal are shown in Fig 12. Except some individual heart sounds, most heart sounds are found to be reduced by 30-50 percent. The filtered results of other typical signals are shown in Fig 13. The results prove the general utilization of the algorithm. Fig 14 and Fig 15 show other filter results of the eleven patients.

## Discussion

The series of algorithms contain three steps of filters, a type I Chebyshev band-pass filter with the pass-band from 20Hz to1600Hz, a modified wavelet threshold de-noising filter and an adaptive filter. Each step of filters processes different components of noises. The FIR band-pass filter reduces most of the high-frequency and low-frequency noises beyond the pass-band, which accounts for the majority of the noises in original signals. However, because of the transitional band and the noise produced by the FIR filter, a single linear filter cannot cancel out all the noises in the stop-band. The modified wavelet threshold filter segments the signal into different frequency regions in the wavelet-domain, and processes the signals in narrower frequency bands to get more effective de-noising results. Majority of the remaining high-frequency and low-frequency noises, a portion of human voices and most of the second heart sounds are reduced in this step. On account of the overlap of heart sounds and lung sound in frequency, the first heart sounds cannot be filtered by the method in frequency domain. The adaptive filter, serving as a time-domain filter, is designed to reduce the first heart sounds. Because the reference is obtained by the estimation of the first heart sounds on the signals to be filtered, no external signals are required in the filter. To attain the trade-off between convergence rate and mean squared error, the step size is chosen as 0.001, and the order of FIR filter is chosen as 32 for reducing the algorithm complexity.

**Figure 16:**
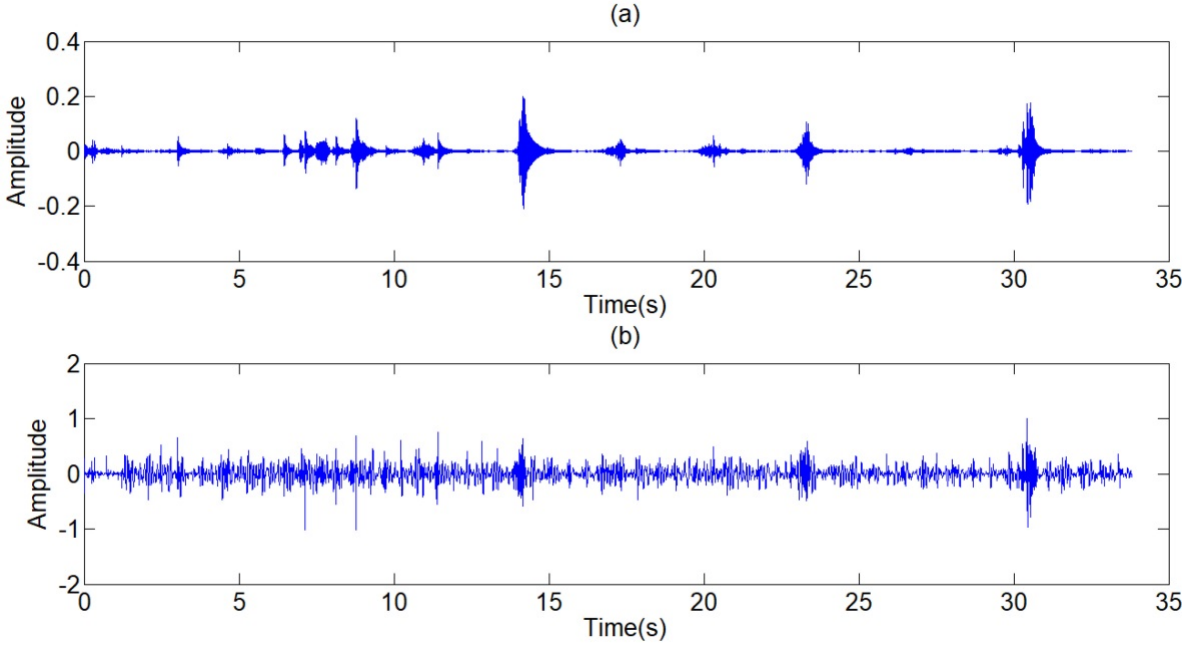
Filtered result of other typical de-noising algorithms. (a) Filtered result of a single FIR band-pass filter with a narrow pass band. (b) Filtered result of the wavelet de-noising algorithm

The filter results of the different signals collected are shown in Fig 12. Judged by the auscultation of physicians, most of the environment noises are canceled out and a large proportion of human voices and heart sounds are reduced, without distortion of the lung sounds. In the aspect of general utilization of algorithm, the frequency domain of pulmonary sounds is relatively stable, the normalization wavelet method based on the power of signal has great improvement in extension of the algorithm, and the algorithm of adaptive filter itself has good universality. Therefore, the series of algorithm has very wide generality and great effect on different signals. The Fig 16 shows the filter result of other typical de-noising algorithms. The part (a) of Fig 16 is the result of a single FIR band-pass filter with a narrow pass band. The algorithm has good effect in de-noising but also leads to much distortion. Fig 4 shows the result of a single FIR band-pass filter with a wide pass band. The signal has no distortion but still has much noise. The part (b) of Fig 16 is the result the traditional wavelet de-noising algorithm. The threshold values are selected by Eq (13). The filtered signal still has too much noise. By comparison, the filter algorithm proposed in this paper shows good effect in de-noising without any distortion.

**Figure 17:**
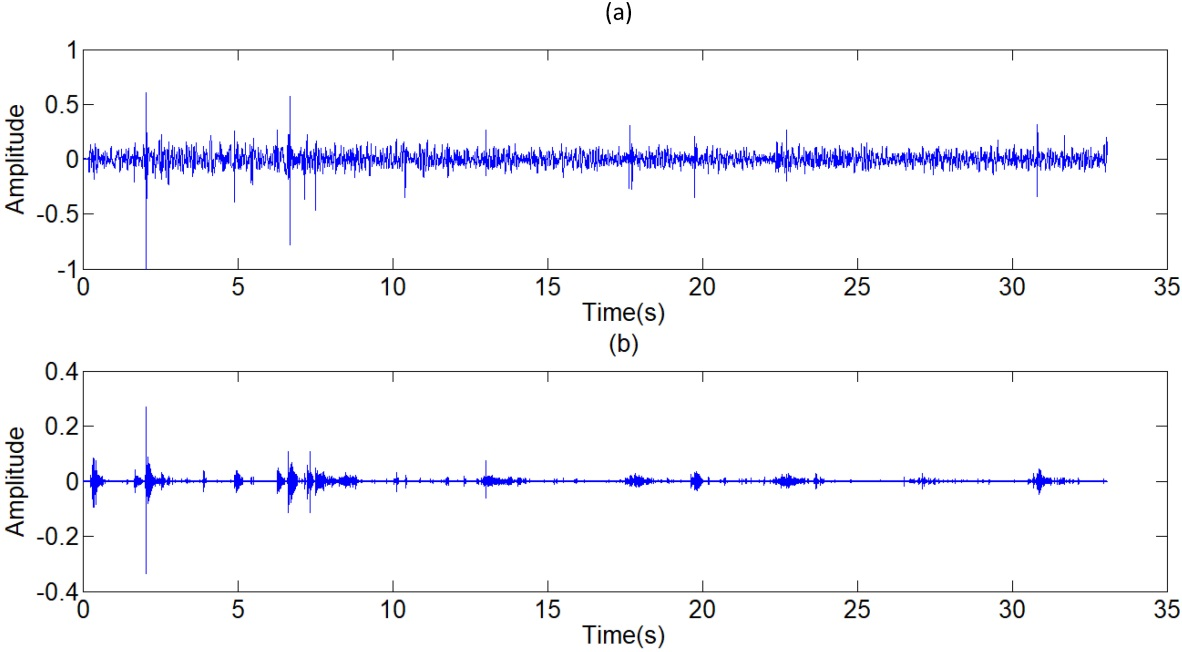
** (a)** Signal with weak acoustic characteristics, **(b)** Filtered result with strengthened components of plosive

Another advantage of the algorithm is to expand signal characteristics of lung sounds. The filter algorithm can be used to expand most acoustic features of different lung sounds, such as plosives in moist rales and wheezes in dry rales. The function of the algorithm can assist physicians to recognize the categories of the respiratory lungs of patients on the basis of acoustic characteristics, and is convenient for the feature detection in the further research of pattern recognition. The function can also make some lung sounds, which are originally not applicable for the week acoustic characteristics, available in the research and auscultation. Because of the occasionally poor signal measuring environment or feebleness of patients, the acoustic characteristics of respiratory sounds are sometimes too week to judge the state of lungs. For instance, the plosives of the moist rale signal shown in Fig 17 are not apparent for the strong environment noises and week components of plosives. The algorithm helps outstanding the plosives, and makes filtered signals contain strong enough components of plosives in the judgment of lung conditions.

## Conclusion

Addressing the issue of suppressing noises in lung sounds, a series of filters are proposed to reduce the external noises and inferences of the collected lung sounds for further analysis. The filter algorithm consists of a Type I Chebyshev band-pass filter, a modified wavelet threshold filter and an adaptive filter. The analysis and the practical experiment show that the filters have good de-noising effect. The algorithm also has a good performance in outstanding the acoustic characteristics of lung sound signals. Adopting the serial integrated de-noising algorithms, the filtered signal can be used to diagnosis better.

## Figures and Tables

**Figure 7:**
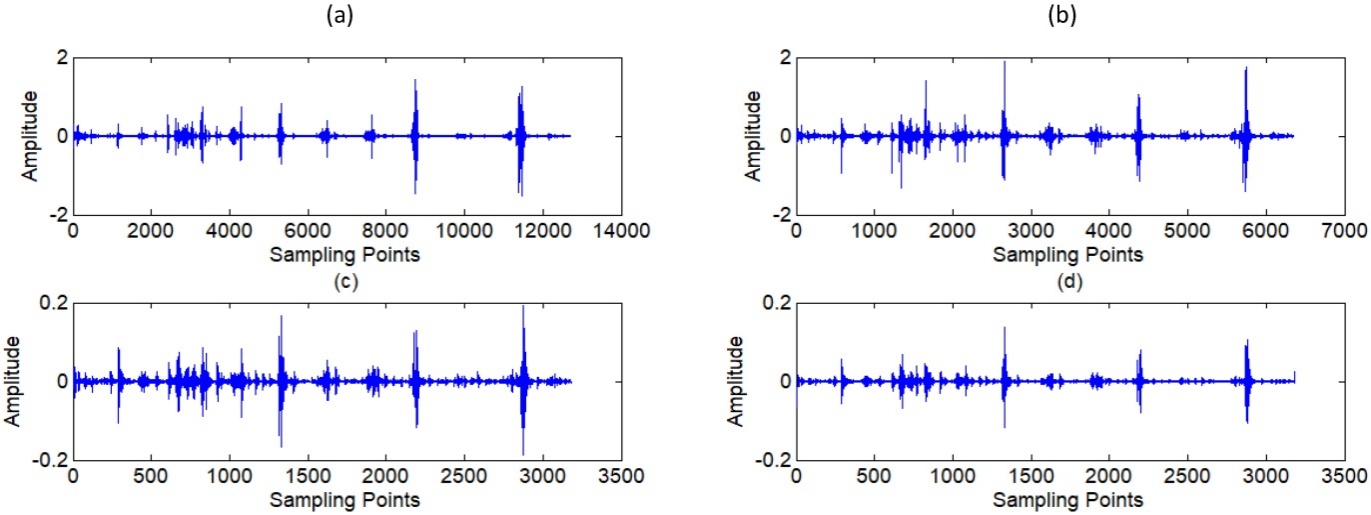
** (a) ~(c)** Wavelet coefficients of standard signal CD7~CD9, **(d)** Approximation coefficients of standard signal CA9

**Table 1:** Selection of threshold type and threshold value

Wavelet Decomposition Layer	Hard Threshold or Soft Threshold (H/S)	Threshold Value
CD1	H	0.01
CD2	H	0.05
CD3	S	0.05
CD4	S	0.001
CD5	S	0.002
CD6	S	0.005
CD9	S	0.08
CA9	S	0.3
